# Reliability and validity of Amharic version of EORTC QLQ-C30 and QLQ-BR23 modules for assessing health-related quality of life among breast cancer patients in Ethiopia

**DOI:** 10.1186/s12955-019-1257-z

**Published:** 2019-12-12

**Authors:** Diriba Alemayehu Gadisa, Esayas Tadesse Gebremariam, Getnet Yimer Ali

**Affiliations:** 1 Lecturer of pharmacology at Pharmacy department, College of Medicine and Health Sciences, Ambo University, Ambo, Ethiopia; 2 Lecturer of pharmacoepidemiology and social pharmacy at Pharmacy department, College of Medicine and Health Sciences, Ambo University, Ambo, Ethiopia; 3 Global One Health initiative, Office of International Affairs, The Ohio State University, Columbus, Ohio USA

**Keywords:** Quality of life, Amharic, Validation, Breast cancer, Ethiopia

## Abstract

**Background:**

Breast cancer is the most common cancer among women and it affects quality of life of those women. So far, the two most frequently used tools for assessing health related quality of life in breast cancer patients, EORTC QLQ-C30 and EORTC QLQ-BR23 modules, were not validated in Ethiopia. Hence, the present study aimed to assess the psychometric properties of the tools among Ethiopian breast cancer patients.

**Methods:**

Institutional based longitudinal study was conducted from January 1 to May 1, 2017 GC at only nationwide oncology center, Tikur Anbessa Specialized Hospital (TASH), Addis Ababa, Ethiopia. A total of 146 patients who visited the facility during that period, with no missing quality of life data, were selected for analysis. The psychometric properties of the EORTC QLQ-C30 and EORTC QLQ-BR23 were evaluated in terms of reliability, convergent, divergent, construct and clinical validity using SPSS version 22.

**Results:**

Satisfactory internal consistency reliability (Cronbach’s α coefficients > 0.7) was confirmed, except for cognitive function (α = 0.516) of EORTC QLQ-C30 and body image (α = 0.510) of EORTC QLQ-BR23. Multiple-trait scaling analysis demonstrated a good convergent and divergent validity. No scaling errors were observed. Most items in EORTC QLQ-BR23 possessed a weak or no correlation with its own dimension in EORTC QLQ-C30 (*r* < 0.4) except with some of symptom scales. A statistically significant chemotherapy induced quality of life scores changes (*P* ≤ 0.05) were observed in all dimensions of both instruments between baseline and the end of first cycle chemotherapy, except for body image (*P* = 0.985) and sexual enjoyment (*P* = 0.817) of EORTC QLQ-BR23, indicating clinical validity.

**Conclusion:**

Amharic version of the EORTC QLQ-C30 and EORTC QLQ-BR23 modules are valid and adequately reliable tool and can be used for clinical and epidemiological cancer researches to study the health related quality of life (HRQoL) of women with breast cancer in Ethiopia.

## Background

According to GLOBOCAN, breast cancer is the most common cancer in women, accounting for 25.1% of all cancers and associated with higher incidence and mortality in developed countries [1]. The impact of cancer on patients’ lives can be measured. Health related quality of life (HRQoL) is defined as a multi-dimensional construct covering disease and treatment-related symptoms, physical, psychological, and social functioning [2, 3].

Measuring HRQoL in cancer treatments is considered as one of the major out-come parameters to measure the efficacy of the chemotherapy in addition to classical biomedical indicators [4–6] which needs reliable and validated instruments in that specific population [3, 7].

European Organization for Research and Treatment of Cancer (EORTC) developed an integrated measurement system for evaluating the quality of life of cancer patients participating in international clinical trials. It includes core European Organization for Research and Treatment of Cancer Quality of Life Questionnaire (EORTC QLQ-C30) and other supplementary modules including breast cancer specific European Organization for Research and Treatment of Cancer Quality of Life Questionnaire (EORTC QLQ-BR 23) [8]. Health related quality of life questionnaire in patients with breast cancer, EORTC QLQ-C30 and EORTC QLQ-BR23, have been developed in English-speaking countries. As a result, its cross socio-cultural and linguistic reliability and validity should be determined for population outside Europe [3, 9, 10]. These measuring instruments have also been translated into different languages world-wide with the support of cross-cultural validation [3].

The only reliability and validity study conducted on the Amharic version of EORTC QLQ-C30 was in gynecological cancer patients by Ayana et al. [11], and this is the first study to asses that among breast cancer patients, in Ethiopia. The earlier study also lacks the inclusion of a construct validity test with other instruments and clinical validity in terms of changes or responsiveness of instruments to clinical changes over a time. Moreover, Amharic version EORTC QLQ-C30 and EORTC QLQ-BR23 questionnaires were not validated for women with breast cancer in Ethiopia though it was previously translated into Amharic [12, 13]. Consequently, the aim of this study is to validate the translated Amharic version of EORTC QLQ-C30 and EORTC QLQ-BR23 in Ethiopian women with breast cancer using a more reliable and informative study design.

### Methods and patients

The institutional based longitudinal study was conducted from January 1 to May 1, 2017 GC at the one and only nationwide oncology center, Tikur Anbessa Specialized Hospital (TASH), Addis Ababa, Ethiopia. We used two time periods (i.e., at baseline and on 21^st^ day of first cycle chemotherapy) and enrolled all the146 patients visited the facility, with no missing quality of life data. A sample size of 100 patients is considered to have enough power to evaluate quality of life study for each specific cancer site [14].Women age above 18 years with proven newly diagnosed breast cancer from stage I to IV and scheduled to receive the most commonly used neo/adjuvant or palliative first line chemotherapy (i.e., Adriamycin-Cyclophosphamide [AC] and Adriamycin-Cyclophosphamide followed by Paclitaxel [AC-T] regimen) were included.

We excluded patients who had previous history of breast cancer treatment (i.e. currently on second line for recurrent breast cancer). Patients with psychiatric disorders, other severe medical illnesses and incomplete quality of life data were also excluded from analysis.

The translation of the EORTC QLQ-C30 and QLQ-BR23 into Amharic version was made considering detailed procedures documented in EORTC Quality of Life Group manual [12, 13, 15]. This study had received the translated Amharic EORTC QLQ-C30 and QLQ-BR23 version from EORTC with permission to use for the proposed study.

Sharp et al. showed that the mode of administering EORTC QLQ-C30 and EORTC QLQ-BR23 questionnaires, whether via interview or self-administration, does not interfere with the scores reported by patients [16]. Unfortunately, in our study, most of the patients had no basic education or had only primary school education, making self-administration of the questionnaire difficult. As a result, all of the study participants were interviewed. Paper based Amharic versions of the EORTC QLQ-C30 and EORTC QLQ-BR23 module were read out loud for patients in a private room by trained oncology nurses. The first interview was made on the same day of their first cycle chemotherapy (i.e., before initiating chemotherapy) whereas the second interview was on first day of their second cycle chemotherapy (i.e., on 21^st^ day) for all patients.

Item scores of the EORTC QLQ-C30 and EORTC QLQ-BR23 were managed according to the EORTC QLQ-C30 scoring manual. After the scoring procedures, all scale and single-item scores were linearly transformed to a 0–100 scale. Higher scores for functional scales and the global quality of life scale, indicate ‘higher level of functioning or global quality of life’, while for symptom scales and single items, a higher score indicates a ‘higher level of symptoms or problems’ [15].

### Statistical analysis

The Statistical Package for Social Sciences (IBM Corp. Released 2013. IBM SPSS Statistics for Windows, Version 22.0, Armon, NY: IBM Corp.) Software was used for analysis. Accordingly, categorical and continuous variables were expressed with frequencies and percentages, and means and standard deviations, respectively.

The psychometric properties of the EORTC QLQ-C30 and EORTC QLQ-BR23 were evaluated in terms of reliability, convergent, divergent, construct and clinical validity test. Reliability (internal consistency) of the questionnaire was tested by Cronbach’s alpha coefficient and the acceptable value to be met was ≥0.70 [8]. Multi-trait scaling analysis was used for item convergent and divergent or discriminant validity. Convergent validity was predicted if the correlation value of an item and its own scale was ≥0.40 and divergent validity if the correlation of an item with its own scale was higher than with other scales. And for clinical validity, quality of life score change over a time was used [8, 17]. As a result, repeated measure ANOVA was used to detect whether there is significant quality of life score change (*P* ≤ 0.05) over a time or not. And a definite scaling success was assumed if the correlation of an item with its own exceeded correlation with other scales [4].

Construct validity was evaluated under the hypothesis that the EORTC QLQ-C30 subscales and EORTC QLQ-BR23 subscales were correlated with each other (acceptable correlation coefficients were ≥ 0.40) [18].

### Ethical issues

The study was approved by Institutional Review Board (IRB) of the school of pharmacy, Collegeof Health Sciences, Addis Ababa University (Ref No:ERB/SOP/09/2016). Informed consent was obtained from all patients prior to participation in the study.

## Results

### Socio-demographic characteristics of the study participants

Among the study participants 98(67.10%) were married, 115(78.80%) had at least one child, 86(58.90%) were house wife, 58(39.70%) were illiterate, and 98(67.10%) were Orthodox. 42.24 years and 25.22Kgm^− 2^ were the mean age and body mass index of the study participants respectively. Majority 131(89.73%) had ductal carcinoma and 135 (92.46%) had Eastern Cooperative Oncology Group (ECOG) performance I (Table 1).

**Table 1:** Socio-demographic data of women with breast cancer at TASH, from January 1 to May 1, 2017 GC, *N* = 146

Category	N (%)	Mean ± SD
Age(Year)		
20–34	30(20.50)	42.24 ± 11.50
35–49	76(52.10)	
50–64	31(21.20)	
≥65	9(6.20)	
Body Mass Index(BMI)(Kgm^−2^)		
< 18.50	14(9.60)	25.22 ± 10.35
18.5–24.99	74(50.70)	
25–29.99	30(23.30)	
≥30	24(16.40)	
Marital status		
Married	98(67.10)	
Widowed	20(13.70)	
Divorced	18(12.30)	
Single	10(6.80)	
Children		
Having child	115(78.80)	
Having no child	31(21.20)	
Occupational status		
House wife	86(58.90)	
Employed	27(18.50)	
Farmer	13(8.90)	
Merchant	13(8.90)	
Daily worker	7(4.80)	
Educational status		
Up to grade 12	63(43.20)	
Illiterate	58(39.70)	
Diploma	15(10.30)	
Degree	10(6.80)	
Religion		
Orthodox	98(67.10)	
Muslim	34(23.30)	
Protestant	14(9.60)	
Histological classification		
Ductal	131(89.73)	
Lobular	6(4.11)	
Mixed	3(2.05)	
Papillary	3(2.05)	
Mucinous	2(1.37)	
Metaplastic	1(0.68)	
Stage		
I	6(4.11)	
II	48(32.87)	
III	64(43.83)	
IV	28(19.17)	
Co morbidity		
Yes	22(15.07)	
No	124(84.93)	
ECOG Performance status		
0	3(2.05)	
I	135(92.46)	
II	5(3.42)	
III	3(2.05)	

*ECOG* European Cooperative Oncology Group, *SD* Standard Deviation

### Reliability test

Table 2 showed that, except for cognitive (α = 0.516) and body image (α = 0.510) scales, all scales had Cronbach’s α coefficients above the acceptable level of 0.70.

**Table 2:** Reliability of EORTC QLQ-C30 and QLQ-BR23 Amharic version in women with breast cancer in Ethiopia (*n* = 146)

EORTC	Scales	Cronbach’s α value^a^
QLQ-C30	Global Health status/QoL	0.789
	Physical functioning	0.771
	Role functioning	0.908
	Emotional functioning	0.817
	Cognitive functioning	0.516
	Social functioning	0.779
	Fatigue	0.851
	Nausea and vomiting	0.857
	Pain	0.739
	Dyspnea	Single item
	Insomnia	Single item
	Appetite loss	Single item
	Constipation	Single item
	Diarrhea	Single item
	Financial difficulties	Single item
QLQ-BR23	Body image	0.510
	Sexual functioning	0.962
	Systemic therapy side effects	0.755
	Breast symptoms	0.773
	Arm symptoms	0.749
	Sexual enjoyment	Single item
	Future perspective	Single item
	Upset by hair loss	Single item

^a^For single items, reliability test is not applicable. EORTC QLQ-C30 and QLQ-BR23 = European Organization for Research and Treatment of Cancer Quality of Life Questionnaire-Core 30 (QLQ-C30) and Breast Cancer specific (QLQ-BR23) questionnaire. QoL = Quality of Life

### Validity test

All item-scale correlation coefficients were above 0.40, except for hair loss which is 0.337, supporting an item convergent validity. Furthermore, the magnitude of the correlation of each item with its own scale exceeded the correlation with another scale and hence it met divergent validity and scaling success (Table 3).

**Table 3:** Multi-trait scale analysis for convergent and divergent validity of EORTC QLQ-C30 and QLQ-BR23 Amharic version in women with breast cancer in Ethiopia (*n* = 146)

EORTC QLQ-C30		Item-own scale correlations^b^	Item-other scale correlations^b^	Scaling success
Scale	Sub-scale			
Overall Quality of life	Global Health Status	0.887–0.909	0.091–0.589	2/2
	Physical	0.442–0.870	0.013–0.572	5/5
	Role	0.958–0.959	0.12–0.639	2/2
Functional scales	Emotional	0.647–0.838	0.008–0.582	4/4
	Cognitive	0.753–0.800	0.025–0.487	2/2
	Social	0.874–0.898	0.041–0.439	2/2
Symptoms scales	Fatigue	0.830–0.880	0.164–0.629	3/3
	Nausea and vomiting	0.769–0.999	0.029–0.377	2/2
	Pain	0.870–0.892	0.116–0.634	2/2
EORTC QLQ-BR23				
Functional scales	Body Image	0.695–0.854	0.026–0.384	4/4
	Sexual functioning	0.961–0.969	0.005–0.029	2/2
Symptoms scales	Therapy’s side effects^a^	0.337–0.7880.636–0.800	0.006–0.534	7/7
	Breast symptoms		0.009–0.636	4/4
	Arm symptoms	0.604–0.856	0.031–0.614	3/3

^a^Systemic therapy side effects, and hair loss correlation with systemic therapy side effect is 0.337 which is below 0.4

^b^Spearman correlation coefficients

### Construct validity

Global Health scale and pain scale were more correlated to symptoms scales of EORTC QLQ-BR23 (r = 0.472–0.553). Fatigue, emotional functioning and cognitive function were moderately correlated with systemic therapy side effects EORTC QLQ-BR23 (r = 0.405–0.633). Out of 45 possible correlations among the scales both questionnaires, 38 were correlated at least at *P* ≤ 0.05 (Table 4).

**Table 4:** Construct validity of EORTC QLQ-C30 Amharic version with its supplementary EORTC QLQ-BR23 scales in women with breast cancer in Ethiopia (*n* = 146)

Spearman’s correlation coefficients					
	Body image	Sexual Functioning	Therapy side effects	Breast symptoms	Arm symptoms
Physical functioning	.224^a^	.174^b^	−.362^a^	−.208^b^	−.329^a^
Role functioning	0.086	.198^b^	−.264^a^	−0.14	−.225^a^
Emotional functioning	.394^a^	0.064	**−.471**^**a**^	−.294^a^	−.328^a^
Cognitive functioning	**.408**^a^	0.125	**−.405**^**a**^	−.177^b^	−.261^a^
Social functioning	.323^a^	.183^b^	−.266^a^	−.216^a^	−.228^a^
Fatigue	−.354^a^	−.245^a^	**.633**^**a**^	.376^a^	**.424**^**a**^
Nausea and vomiting	−.197^b^	0.005	.302^a^	.186^b^	.262^a^
Pain	−.214^a^	−0.06	**.516**^**a**^	**.456**^**a**^	**.495**^**a**^
Global Health score	.387^a^	0.15	**−.553**^**a**^	**−.472**^**a**^	**−.484**^**a**^

^a^Correlation is significant at the 0.01 level (2-tailed)

^b^Correlation is significant at the 0.05 level (2-tailed)

The bold entries indicate those sub-scales having spearman's correlation coefficients≥0.40

### Clinical validity

Repeated measures ANOVA showed all EORTC QLQ-C30 scales changed significantly over a time (*P* < 0.05). It also showed significant change of EORTC QLQ-BR23 over a time except for body image and sexual enjoyment. And all significant changes were towards expected direction (Table 5).

**Table 5:** Clinical validity EORTC QLQ-C30 and QLQ-BR23 Amharic version in women with breast cancer in Ethiopia (*n* = 146)

Quality of life score: mean(SD)				
	EORTC-QLQ-C30	Baseline	End of 1st CT^b^ cycle	*p*-value
Overall QoL	Global Health status	57.36(20.09)	41.72(19.24)	0.000
	Physical	68.63(26.06)	51.00(23.70)	0.000
	Role	52.74(38.85)	23.74(31.37)	0.000
Functional scales	Emotional	80.02(22.60)	67.81(25.14)	0.000
	Cognitive	86.41(19.96)	77.39(26.31)	0.000
	Social	74.88(30.59)	58.22(33.11)	0.000
	Fatigue	28.69(30.40)	72.07(26.10)	0.000
Symptoms scales	Nausea and Vomiting	3.31(11.84)	53.53(34.19)	0.000
	Pain	32.53(30.53)	42.00(33.11)	0.001
	Dyspnea	19.40(29.75)	28.76(29.97)	0.003
	Insomnia	27.85(34.58)	35.61(37.88)	0.024
Single items	Appetite loss	17.80(29.59)	71.23(31.22)	0.000
	Constipation	10.27(21.99)	22.83(31.74)	0.000
	Diarrhea	2.74(12.07)	16.89(30.38)	0.000
	Financial problem	61.18(36.72)	65.52(33.77)	0.000
EORTC-QLQ-BR23				
Functional scales	Body Image	72.54(42.45)	72.60(27.24)	0.985
	Sexual Functioning	21.11(36.95)	12.21(28.72)	0.004
	Therapy^a^ side effects	12.88(15.08)	50.71(19.70)	0.000
Symptoms scale	Breast symptoms	22.48(22.34)	17.63(19.94)	0.000
	Arm symptoms	26.03(26.08)	18.79(23.18)	0.000
Single items	Future perspectives	69.63(33.43)	63.69(35.21)	0.041
	Sexual Enjoyment	72.91(32.70)	70.83(34.15)	0.817

^a^Systemic therapy side effects

^b^After three weeks, on 21^st^ day of the CT (Chemotherapy) or on the first day of 2^nd^ cycle CT.

*SD* Standard Deviation, *QoL* Quality of Life

## Discussions

Health-related quality of life is considered as an important endpoint in cancer clinical trials [2]. There is a need to complement conventional clinical outcomes with information representing the patients’ perception of outcome. A better understanding of HRQoL may lead to enhanced care of patients with cancer [17].

The measurement of patient-reported outcomes, including health-related quality of life, with reliable and valid tools and its incorporation into clinical practice for breast cancer patients have paramount advantages [2, 7]. EORTC QLQ-C30 HRQoL scales provide valuable prognostic information when combined with socio-demographic and clinical information [3].

Our study showed that Amharic version EORTC QLQ-C30 instrument was reliable as the value for internal consistency ranges from 0.739–0.908 except for cognitive domain (α = 0.516) which had Cronbach’s alpha value ≤0.7. Different studies in different parts of the world, Albanian (α = 0.54) [18], Taiwan (α = 0.54) [19], Thailand (α = 0.50) [4], Arabic (α = 0.67) [10], Amharic version in gynecological cancer in Ethiopian (α = 0.29) [11], Mexican-Spanish (α = 0.52) [17], Moroccan (α = 0.34) [5], Singapore (α = 0.19) and others reviewed by Luo et al. [20] showed as cognitive functioning did not meet the internal consistency standards.

From EORTC QLQ-BR23, body image showed minimum internal consistency (α = 0.510) which was below the minimum requirements (α ≤ 0.7) in contrast to systemic side effects among Iranian patients (α = 0.63) [9]. However, other scales showed good internal consistency which ranges from 0.749–0.962. Our study, however, showed the lower internal consistency value compared with the Spanish breast cancer patients (0.46–0.94) while it was higher when compared with the result from the American and Dutch breast cancer patients (0.57–0.91) [21]. Role functioning (α = 0.908), nausea and vomiting (α = 0.857) and fatigue (α = 0.851) from EORTC QLQ-C30 and sexual functioning (α = 0.962) from EORTC QLQ-BR23 showed strong internal consistency. The Brazilian version also showed higher scores in both instruments (α = 0.72–0.86) [22].

In our study, multi-trait scaling analysis showed that almost all of items had strong correlations with their respective sub-scale (r ≥ 0.6) that indicates a strong convergent validity of the instruments [17], with the exception of hair loss to systemic therapy side effects. There was no scaling error found as all of the items of both instruments discriminate significantly between their own and other domains indicating divergent validity. This is in contrast to previous study in patients with gynecological cancer for EORTC QLQ-C30 [11]. Our study was consistent with different studies conducted elsewhere [5, 10, 20, 23]. As a result, the translated Amharic version EORTC QLQ-C30 [13] and EOTRC QLQ-BR23 [12] were psychometrically valid in Ethiopian women with breast cancer.

There were strong correlations (r = 0.405–0.633) between systemic therapy side effects of EORTC QLQ-BR23 and emotional, cognitive, fatigue, pain and global health status of EORTC QLQ-C30. Arm symptoms also showed strong correlations (r = 0.424–0.495) with fatigue, global health status and pain whereas breast symptoms showed moderate correlations with pain and global health status. This implies that symptom scales of EORTC QLQ-BR23 were more correlated with the corresponding scales of EORTC QLQ-C30 than the functional scales. Furthermore, our study showed significant correlations (*P* ≤ 0.05) between the two instruments’ scales but the majority showed weak (*r* < 0.4) or no significant correlations, in particular sexual functioning, with other domains. Weak or no correlation indicates the EORTC QLQ-BR23 has unique domains of HRQoL, which are not addressed by the EORTC QLQ-C30. As a result, this finding further strengthens as EORTC QLQ-BR23 only used with EORTC QLQ C-30 [21] to assess the HRQoL of breast cancer patients in different way to EORTC QLQ-C30.

Health-related quality of life is a dynamic multidimensional measurement that changes over time and within the same patient [17]. Likewise, except for body image (*P* = 0.985) and sexual enjoyment (*P* = 0.817) of EORTC QLQ-BR23, all sub-scales and single items of EORTC QLQ-C30 and EORTC QLQ-BR23 quality of life scores showed significant chemotherapy induced changes (*P* ≤ 0.05) between pretreatment and on 21^st^ day of first cycle chemotherapy. The changes were towards the expected direction. Functional and Global quality of life domains scores were decreased while the symptom scales were increased. These indicates deterioration of quality of life from the baseline [15]. Hence, both questionnaires effectively discriminate quality of life scores at different point in a time which ensures clinical validity of the instruments.

Our study also had some limitations as we didn’t manage to measure test–retest reliability of the EORTC QLQ-C30 and EORTC QLQ-BR23. And we didn’t check the external convergent validity, the gold standard test to assess validity, due mainly to unavailability of other validated HRQoL assessment tool. We have tried to overcome those limitations by checking construct validity and responsiveness of both instruments over a time.

## Conclusions

In general, the translated Amharic version of EORTC QLQ-C30 and EORTC QLQ-BR23 met satisfactory reliability standards, and convergent, divergent, construct and clinical validity to be used both in research and clinical as a measure of treatment out-come with respect to quality of life in women with breast cancer in Ethiopia.

## Data Availability

Please contact the first author for data requests.

## Abbreviations

ECOG: European Cooperative Oncology Group
EORTC QLQ-BR23: European Organization for Research and Treatment of Cancer Quality of Life Questionnaire breast cancer specific module
EORTC QLQ-C30: European Organization for Research and Treatment of Cancer Quality of life Questionnaire version 3
HRQoL: Health Related Quality of life; TASH: Tikur Anbessa Specialized Hospital
